# Validity of a 10-Item Menstrual Quality-of-Life Measure in Women With Heavy Menstrual Bleeding

**DOI:** 10.7759/cureus.116957

**Published:** 2026-09-26

**Authors:** Hasina Sadiq, Shafaq Fatima, Filza Sibtain, Maria Aslam, Mahrukh Zaidi

**Affiliations:** 1 Obstetrics and Gynecology, Nottingham University Hospitals NHS Trust, Nottingham, GBR; 2 Obstetrics and Gynecology, Kahuta Research Laboratories (KRL) Hospital, Islamabad, PAK; 3 Obstetrics and Gynecology, Shifa International Hospitals Limited, Islamabad, PAK; 4 Gynecologic Oncology, Shaukat Khanum Memorial Cancer Hospital and Research Centre, Lahore, PAK; 5 Surgical Oncology, Shaukat Khanum Memorial Cancer Hospital and Research Centre, Lahore, PAK

**Keywords:** heavy menstrual bleeding (hmb), pbac, period-qol, quality of life, validation

## Abstract

Background and aim

Heavy menstrual bleeding, which is a common gynecological condition, has a detrimental effect on a woman's physical, psychological, and social well-being. With its high prevalence, it is underdiagnosed due to the reliance on subjective assessment and the absence of validated tools in many populations. A 10-item questionnaire was used to assess the impact of menstrual symptoms on quality of life (QOL). Few tools evaluate the effect of heavy menstrual bleeding on quality of life using the pictorial blood loss assessment chart (PBAC) and assess the validity of a translated menstrual quality-of-life questionnaire in the local population. This study aimed to determine the effect of heavy menstrual bleeding (HMB), using the PBAC, on women's quality of life and to assess the validity of the translated version of the menstrual quality-of-life questionnaire in our population.

Methods

A prospective cross-sectional study was conducted over a period of six months in the gynecology outpatient department. A total of 330 women aged 18-50 years diagnosed with heavy menstrual bleeding (HMB) were recruited through non-probability consecutive sampling. HMB was defined as a PBAC score ≥100 supported by hemoglobin levels. Demographic variables, clinical features, and the translated 10-item questionnaire were included in the structured proforma used for data collection. Statistical analyses were performed using SPSS version 23 (Armonk, NY: IBM Corp.).

Results

On average, participants were 40.25±5.69 years old, with a hemoglobin level of 9.80±1.22 g/dL and a PBAC score of 266.04±86.21. The most prevalent cause of HMB was leiomyoma/fibroid, accounting for 51.8%. The mean quality-of-life score was 35.16±4.06, suggesting considerable detriment to quality of life. A severe impairment was observed in 80.9% of women. The translated PERIOD-QOL tool demonstrated acceptable internal consistency (Cronbach's alpha=0.606). A weak but statistically significant positive correlation was observed between PBAC scores and quality-of-life scores (rho=0.167, p=0.002), suggesting that increased menstrual blood loss is associated with poorer quality of life.

Conclusion

Heavy menstrual bleeding has a significant adverse impact on quality of life among women. The translated 10-item questionnaire demonstrated acceptable internal consistency for assessing quality-of-life impairment in the local population and may facilitate early identification and management of affected women.

## Introduction

Heavy menstrual bleeding (HMB) is defined as excessive menstrual blood loss that interferes with a woman's quality of life. It is one of the most common complaints presenting to gynecology and obstetrics outpatient departments [1]. In addition to adversely affecting quality of life, HMB imposes a considerable economic burden on affected women and healthcare systems [2].

Despite its substantial physical and psychological impact, gaps remain between women's knowledge and understanding of HMB and its potential consequences [3]. Up to one-third of women of reproductive age experience HMB. A multinational study conducted across South Asia and sub-Saharan Africa reported a prevalence of HMB of 48.6% [4]. In Pakistan, the prevalence of HMB has been reported to be approximately 37.9% among women of reproductive age [5].

HMB has traditionally been described as menstrual blood loss exceeding 80 mL per menstrual cycle [6]. However, quantitative assessment of menstrual blood loss is primarily useful in research settings, as accurately measuring menstrual blood loss in clinical practice is difficult. HMB remains underdiagnosed and undertreated, partly because of the poor correlation between patients' perceptions and objectively measured menstrual blood loss and the limited availability of validated diagnostic tools [7,8]. Pictorial blood loss assessment charts (PBACs) have been developed to estimate menstrual blood loss and have been used in both research and clinical settings [9]. A study conducted in Hong Kong demonstrated the utility of the PBAC for self-assessment of menstrual blood loss and for differentiating normal menstruation from HMB among Asian women [10].

Women's subjective assessment alone may not reliably quantify menstrual blood loss because it requires accurate recall and recording of menstrual characteristics across individual cycles. Therefore, clinical assessment should focus not only on the perceived amount of bleeding but also on its impact on daily functioning and quality of life. The National Institute for Health and Care Excellence (NICE) recommends discussing the woman's symptoms and the impact of bleeding on her quality of life rather than relying solely on an objective estimate of blood loss [11]. Brief validated screening tools, including the six-item SAngrado Menstrual AbundaNTe en ginecologíA (SAMANTA) questionnaire and a two-item visual analog scale, have been developed to facilitate the identification of HMB that interferes with quality of life in clinical practice [8,12].

More recently, a brief menstrual quality-of-life measure has been developed to assess the impact of HMB on women's quality of life [13]. Such patient-reported outcome measures may facilitate recognition of the burden of HMB and encourage women to seek timely medical care, potentially contributing to earlier diagnosis and appropriate treatment.

## Materials and methods

This prospective cross-sectional study was conducted in the gynecology outpatient departments of Shifa College of Medicine/Falahi Foundation and Kahuta Research Laboratories (KRL) Hospital, Islamabad, over a period of six months after approval from the institutional ethics committees of both hospitals. The sample size was calculated using the World Health Organization (WHO) sample size calculator with a 95% confidence level, an anticipated HMB prevalence of 31.3%, and an absolute precision of 5%; the resulting sample size was 330 participants.

Women attending the gynecology outpatient clinics were recruited using non-probability consecutive sampling according to predefined eligibility criteria. The inclusion and exclusion criteria are summarized in Table 1. All eligible women were briefed about the study, and written informed consent was obtained. HMB was defined by a PBAC score ≥100 and supported by hemoglobin assessment. The PBAC was originally described by Higham et al. in 1990 [14]. It provides a semiquantitative assessment of menstrual blood loss using standardized scoring of sanitary pads and tampons, the degree of staining, clot size, and flooding episodes. A PBAC score of 100 or more was used to identify HMB. Hemoglobin measurement within the preceding three months was also recorded.

**Table 1:** Inclusion and exclusion criteria.

Inclusion criteria	Exclusion criteria
Premenopausal women aged 18-50 years	Premature ovarian insufficiency
Attending the gynecology outpatient department	Postmenopausal women, including surgical menopause
Heavy menstrual bleeding	Pregnant or lactating women
Able to understand and speak Urdu	Language barrier preventing understanding of the questionnaire
Able to provide informed consent	Unable to provide informed consent

Data were collected using a structured, self-administered proforma. Part A recorded baseline demographic and clinical variables, including age, body mass index (BMI), years of marriage, parity, cause of HMB according to the International Federation of Gynecology and Obstetrics (FIGO) PALM-COEIN classification, and PBAC score [15]. Part B contained the translated version of the 10-item Period Quality of Life (PERIOD-QOL) questionnaire developed by Lancastle et al. [13]. The original PERIOD-QOL was developed as a brief self-administered measure of menstrual quality of life (QOL). The questionnaire was translated into Urdu by two study authors and was pilot-tested in 10 women with HMB and 10 women from the general population to assess comprehensibility. The original PERIOD-QOL publication is available under a Creative Commons Attribution 4.0 International License; we maintained appropriate attribution. Statistical analyses were performed using Statistical Package for the Social Sciences (SPSS), version 23 (Armonk, NY: IBM Corp.). Continuous variables were summarized using means and standard deviations (SDs) and medians with interquartile ranges (IQRs), as appropriate. Spearman's rank-order correlation was used to assess associations between PBAC score, hemoglobin, age, and PERIOD-QOL score. The Kruskal-Wallis test was used to compare PERIOD-QOL scores across HMB etiological groups. A two-sided p<0.05 was considered statistically significant.

## Results

A total of 330 respondents with heavy menstrual bleeding (HMB) were included in the analysis. After data screening, one implausible age entry of 5.8 years was treated as missing for age-related summaries. The mean age was 40.25±5.69 years, the mean hemoglobin level was 9.80±1.22 g/dL, and the mean pictorial blood loss assessment chart (PBAC) score was 266.04±86.21. The mean Period Quality of Life (PERIOD-QOL) total score was 35.16±4.06. Baseline and clinical characteristics are presented in Table 2.

**Table 2:** Baseline and clinical characteristics. IQR: interquartile range; PBAC: pictorial blood loss assessment chart; PERIOD-QOL: Period Quality of Life

Variables	n	Mean	SD	Median (IQR)	Range
Age (years)	329	40.25	5.69	40 (37-44)	20-51
Parity	330	3.19	1.81	3 (2-4)	0-8
Number of children	330	3.19	1.81	3 (2-4)	0-8
Hemoglobin (g/dL)	330	9.8	1.22	10 (9-11)	7-13
PBAC score	330	266.04	86.21	250 (200-330)	102-480
PERIOD-QOL total score	330	35.16	4.06	36 (33-38)	18-45

Most women fell in the severe impact category (267, 80.9%), with 38 (11.5%) in the moderate impact category and 23 (7.0%) in the very low impact category. There was a weak but statistically significant positive correlation between PBAC score and PERIOD-QOL total score (Spearman's rho=0.167, p=0.002), indicating that higher menstrual blood loss was associated with poorer quality of life. Hemoglobin showed a weak inverse, non-significant correlation with PERIOD-QOL score (Spearman's rho = -0.094, p=0.090). The distribution of quality-of-life impact categories is shown in Figure 1. The translated PERIOD-QOL scale showed acceptable internal consistency, with a Cronbach's alpha of 0.606. Item-wise responses to the 10-item PERIOD-QOL questionnaire are presented in Table 3.

**Figure 1:**
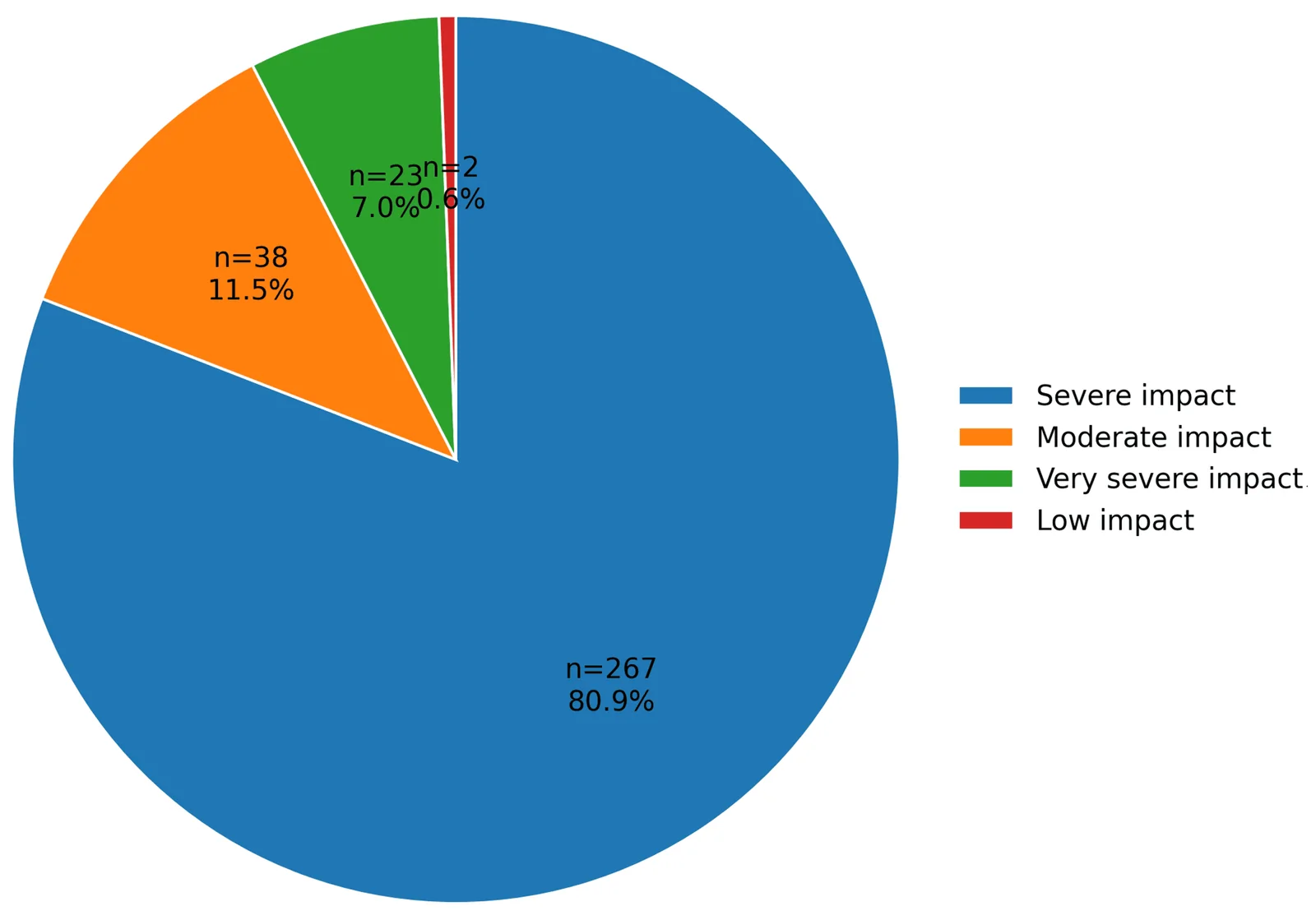
Distribution of QOL impact categories. QOL: quality of life

**Table 3:** Item-wise PERIOD-QOL responses. n: number of participants; Q1-Q10: questions 1-10; PERIOD-QOL: Period Quality of Life

Item	Questions	Mean	SD	Most common response	n	%	Not at all	A little bit	Somewhat	A great deal	A very great deal
Q1	Periods bothered because extremely heavy	4.12	0.7	A great deal	178	53.9	0	5	49	178	98
Q2	Worried about leaking or soiling of clothes	3.62	0.96	A great deal	161	48.8	10	33	77	161	49
Q3	Severe pain during periods	3.42	0.84	A great deal	149	45.2	6	38	117	149	20
Q4	Heavy periods make me worry about my health	3.76	1.04	Somewhat	113	34.2	3	32	113	74	108
Q5	Periods make me miss work	3.51	0.81	A great deal	189	57.3	3	43	81	189	14
Q6	Periods make me feel tired and upset	3.59	0.82	A great deal	204	61.8	11	20	78	204	17
Q7	Periods affect my sex life	3.18	1.06	A great deal	129	39.1	36	35	111	129	19
Q8	Periods interfere with exercise, vacations	3.18	0.78	Somewhat	195	59.1	8	34	195	76	17
Q9	Prolonged cycles than other females	3.39	0.79	A great deal	172	52.1	8	36	110	172	4
Q10	Worried before start of periods	3.38	0.77	A great deal	153	46.4	4	38	126	153	9

There was a weak but statistically significant positive correlation between PBAC score and PERIOD-QOL total score (Spearman's rho=0.167, p=0.002), indicating that higher menstrual blood loss was associated with poorer quality of life. Hemoglobin showed a weak inverse, non-significant correlation with PERIOD-QOL score (Spearman's rho = -0.094, p=0.090). Detailed correlation findings are presented in Table 4.

**Table 4:** Correlations between pictorial blood loss assessment chart (PBAC), hemoglobin, age, and quality-of-life scores n: number of participants; PBAC: pictorial blood loss assessment chart; PERIOD-QOL: Period Quality of Life; rho: Spearman's rank correlation coefficient

Variable pairs	n	Spearman's rho	p-Value
PBAC score vs. PERIOD-QOL total score	330	0.167	0.002
Hemoglobin vs. PERIOD-QOL total score	330	-0.094	0.090
Age vs. PERIOD-QOL total score	329	0.107	0.052
PBAC score vs. hemoglobin	330	0.062	0.262

Mean PERIOD-QOL scores did not differ significantly across the etiological groups of HMB (Kruskal-Wallis p=0.332). The PERIOD-QOL scores by cause of HMB are presented in Table 5, and the corresponding mean scores are illustrated in Figure 2.

**Table 5:** PERIOD-QOL score by cause of HMB. HMB: heavy menstrual bleeding; IQR: interquartile range; PERIOD-QOL: Period Quality of Life

Cause of HMB	n	Mean QOL score	SD	Median (IQR)	Range	Kruskal-Wallis p-value
Leiomyoma/fibroid	171	34.94	4.3	36 (33-37)	18-45	0.332
Endometrial	65	35.12	4.06	36 (33-38)	23-45	
Ovulatory disorder	36	34.75	3.31	36 (33-37)	26-40	
Polyp	26	36.62	3.48	37 (35-38)	26-42	
Adenomyosis	18	35.39	4.27	36 (33-39)	28-42	
Malignancy	14	36.07	3.29	37 (34-38)	29-41	

**Figure 2:**
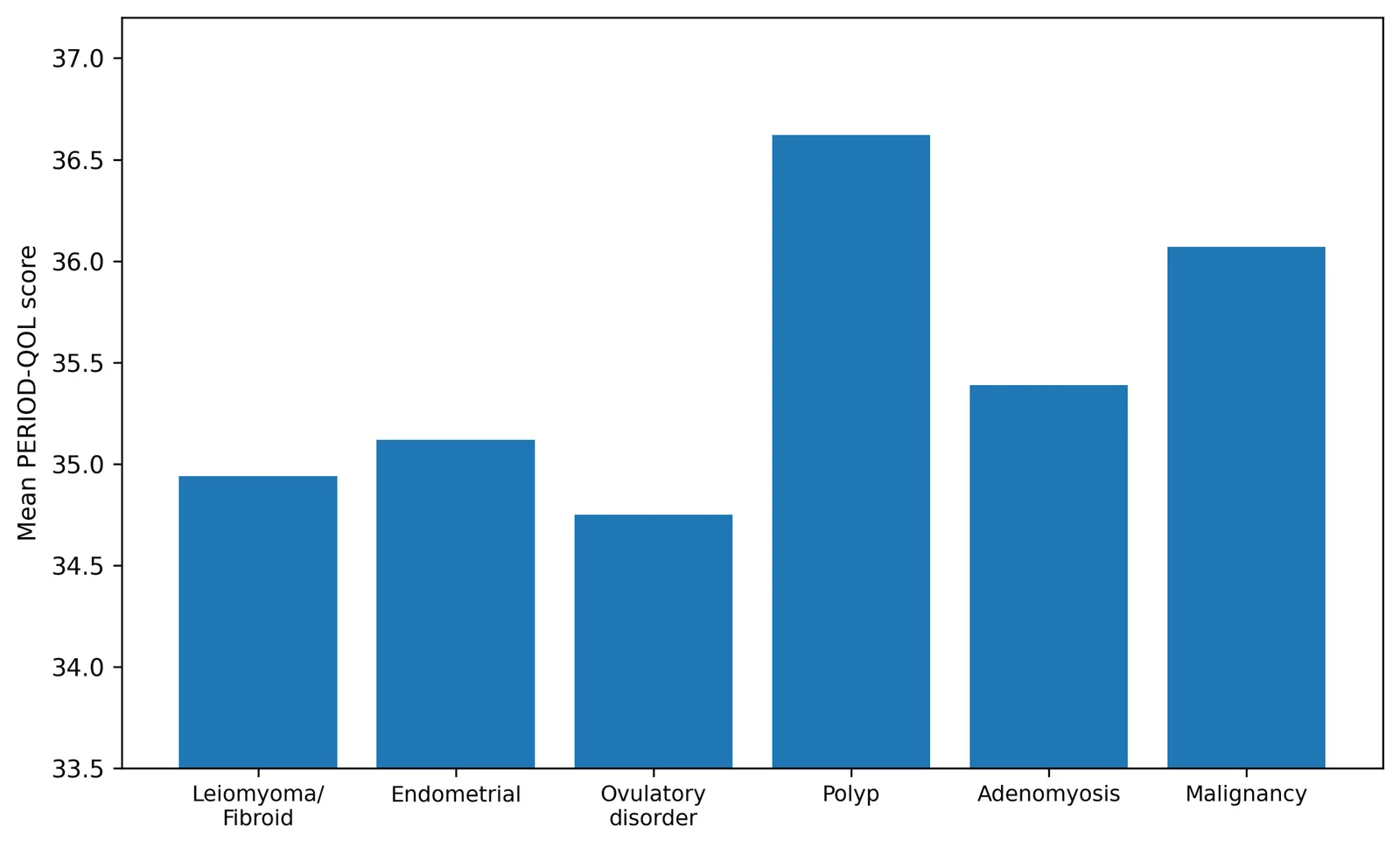
Mean PERIOD-QOL score by cause of HMB. HMB: heavy menstrual bleeding; PERIOD-QOL: Period Quality of Life

## Discussion

This study assessed the impact of HMB on QOL using a translated tool, PERIOD-QOL, and explored its validity in the Pakistani population. The study demonstrates that HMB has a considerable negative impact on women's quality of life, with the majority experiencing severe impairment. Moreover, the PERIOD-QOL scale showed acceptable internal consistency and was significantly correlated with menstrual blood loss as measured by the PBAC score.

In this study, the mean PERIOD-QOL score (35.16±4.06) reflects an associated substantial deterioration in quality of life, which is corroborated by a similar conclusion found in most previous literature identifying HMB with considerable burden on physical, psychological, and social behavior [14]. Fraser et al. and Bitzer had previously reported a heavy impact with regard to daily functioning, work productivity, psychological well-being, and physical activity [15,16]. The large proportion of women in the severe impact category (80.9%) noted in our study adds to evidence of the scale of this problem occurring in low- and middle-income settings, where awareness and healthcare-seeking behavior may be limited.

Our results are also in line with multinational data published by Sinharoy et al., who demonstrated that HMB was highly prevalent and strongly associated with reduced well-being in places with few resources [4].⁠ As per local data, a significant percentage of Pakistani women are affected by HMB, which frequently results in anemia and fatigue [5]. The mean hemoglobin level in our study (9.8 g/dL) reflects the clinical burden of chronic blood loss, although its correlation with QOL was weak and not statistically significant. This implies that the subjective perception of symptoms may be a more significant factor in determining quality of life than hemoglobin levels alone.

The small but statistically significant positive correlation between PBAC scores and PERIOD-QOL scores (rho=0.167, p=0.002), which suggests that increased periodic blood loss is linked to declining quality of life, is one of the study's main findings. This is consistent with other research showing a correlation between symptom severity and patient-reported outcomes and objective measurements of blood loss, such as PBAC [17]. The comparatively small link, however, emphasizes the complexity of HMB, where sociocultural variables, coping strategies, and individual perspectives all play significant roles.

Remarkably, there was no discernible variation in QOL scores among the various etiological sources of HMB (p=0.332). This implies that women's subjective burden is similar independent of underlying illness, such as leiomyoma, endometrial causes, or ovulatory abnormalities. Similar findings have been documented in previous research, where the main factor influencing quality of life impairment was symptom severity rather than etiology [6].

Moderate reliability for exploratory clinical use was indicated by the translated PERIOD-QOL tool's acceptable internal consistency (Cronbach's alpha=0.606). This value is within an acceptable range for newly adapted instruments in various cultural contexts, even though it is marginally lower than that reported in the initial validation research by Lancastle et al. [13]. Since cultural views on menstruation and symptom reporting might affect responses, translation and local validation are crucial processes. The findings suggest that the tool can be used as a screening instrument in outpatient settings to identify women with significant QOL impairment.

Item-wise analysis revealed that the most affected domains included heavy bleeding, fear of leakage, fatigue, and absenteeism from work. These findings are consistent with previous studies, which have demonstrated that HMB commonly leads to social embarrassment, reduced physical activity, and occupational limitations [16]. The impact on sexual life and emotional well-being further underscores the multidimensional burden of the condition.

This study has important clinical implications. Given the poor correlation between objective blood loss and patient perception, as highlighted in previous literature, reliance solely on quantitative measures may lead to underdiagnosis [18,19]. Incorporating brief, validated QOL tools such as PERIOD-QOL into routine clinical practice can facilitate early identification of affected women and improve patient-centered care, in line with National Institute for Health and Care Excellence (NICE) recommendations [20].

It should also be acknowledged that new information is available related to HMB-specific instruments to assess quality of life [21]. In a recent systematic review and critical appraisal published in 2025, Al Wattar et al. evaluated 10 disease-specific QOL instruments for HMB [21]. They found that some show promise, but none meet all methodological quality criteria recommended by the Consensus-based Standards for the Selection of Health Measurement Instruments (COSMIN) framework for the time being. The researchers mentioned that further validation research in different setting and populations is needed. This study contributes to the existing evidence base by providing one of the first assessments of the translated PERIOD-QOL questionnaire in a South Asian population and showing its strong association with objectively measured menstrual blood loss.

This study has a few limitations. Due to its cross-sectional design, it is impossible to establish causality. The use of non-probability sequential sampling may create selection bias. The internal consistency of the tool was moderate and acceptable, but may need further refinement and validation within a larger and more diverse population. In addition, recall and reporting biases may affect self-reported measures.

## Conclusions

Heavy menstrual bleeding was linked to a significant negative impact on quality of life, with the majority of women suffering from severe impairment. The translated PERIOD-QOL questionnaire supported its potential use as an additional tool for identifying women with significant quality of life impairment by demonstrating acceptable internal consistency and a weak but statistically significant association with objectively assessed menstrual blood loss using the PBAC score. However, before widespread clinical use, additional validation in larger and more diverse groups is necessary due to the cross-sectional design, non-probability sampling, and moderate internal consistency.
